# LINC02418 promotes colon cancer progression by suppressing apoptosis via interaction with miR-34b-5p/BCL2 axis

**DOI:** 10.1186/s12935-020-01530-2

**Published:** 2020-09-22

**Authors:** Jun Tian, Peng Cui, Yifei Li, Xuequan Yao, Xiaoyu Wu, Zhirong Wang, Chunsheng Li

**Affiliations:** 1 Department of General Surgery, Zhangjiagang Traditional Chinese Medicine Hospital, Affiliated to Nanjing University of Chinese Medicine, Zhangjiagang, 215600 Jiangsu China; 2 Department of Gastrointestinal Surgery, Shanghai Pudong Hospital, Fudan University Pudong Medical Center, Shanghai, 201399 China; 3 Department of Geriatrics, The Affiliated Hospital to Changchun University of Chinese Medicine, Changchun, 130021 Jilin China; 4 Department of Gastrointestinal Surgery, The Affiliated Hospital of Nanjing University of Chinese Medicine, Nanjing, 210029 Jiangsu China; 5 Department of Orthopaedics, Zhangjiagang Traditional Chinese Medicine Hospital, Affiliated to Nanjing University of Chinese Medicine, Zhangjiagang, 215600 Jiangsu China; 6 Department of Gastrointestinal Colorectal and Anal Surgery, China-Japan Union Hospital of Jilin University, No. 126 Xiantai Street, Changchun, 130033 Jilin China

**Keywords:** LINC02418, Colorectal cancer, BCL2, microRNA-34b-5p, ceRNA

## Abstract

**Background:**

LncRNAs act as functional regulators in tumor progression through interacting with various signaling pathways in multiple types of cancer. However, the effect of LINC02418 on colorectal cancer (CRC) progression and the underling mechanisms remain unclear.

**Methods:**

LncRNA expression profile in CRC tissues was investigated by the TCGA database. The expressional level of LINC02418 in CRC patients was determined by quantitative reverse transcription-polymerase chain reaction (qRT-PCR). Kaplan–Meier analyses was used to investigate the correlation between LINC02418 and overall survival (OS) of CRC patients. Cell proliferative, migratory and invasive abilities were detected by CCK-8 assays, colony formation assays and trans-well assays in HCT116 and LoVo cells which were stably transduced with sh-LINC02418 or sh-NC. The binding between LINC02418 and miR-34b-5p, and the interaction between miR-34b-5p and BCL2 were determined by dual-luciferase assays. Western blot experiments were conducted to further explore the effect of miR-34b-5p on BCL2 signaling pathway. Rescue experiments were performed to uncover the role of LINC02418/miR-34b-5p/BCL2 axis in CRC progression.

**Results:**

LINC02418 was upregulated in human colon cancer samples when compared with adjacent tissue, and its high expressional level correlated with poor prognosis of CRC patients. LINC02418 promoted cancer progression by enhancing tumor growth, cell mobility and invasiveness of colon cancer cells. Additionally, LINC02418 could physically bind to miR-34b-5p and subsequently affect BCL2 signaling pathway. Down-regulation of LINC02418 reduced cell proliferation, while transfection of miR-34b-5p inhibitor or BCL2 into LINC02418-silenced CRC cells significantly promoted CRC cells growth.

**Conclusions:**

LINC02418 was upregulated in human CRC samples and could be used as the indicator for prediction of prognosis. LINC02418 acted as a tumor driver by negatively regulating cell apoptosis through LINC02418/miR-34b-5p/BCL2 axis in CRC.

## Background

Colorectal cancer (CRC), also known as colon cancer, is one of the principle malignancies worldwide. Although improvements have been made in the diagnosis and treatment, CRC remains the top leading cause of cancer-related death [1, 2]. The metastasis usually occurs in the late stage of CRC in which tumor cells detach from the primary tumor, invade into surrounding tissue or vessels, migrate and colonize at distant organs such as liver and lung. Metastasis is the main cause of CRC-related death, thus uncovering the molecular mechanisms and identification of new diagnostic markers are in emerging need during current CRC studies. The progress of normal intestinal epithelial cell transition to unregulated cancer cell is a multi-stage and complicated process which is associated with the accumulation of both genetic and epigenetic changes. The aberration, mutations of oncogenes or tumor suppressive genes, and epigenetic alterations all lead to the progression of CRC [3–6].

Long non-coding RNAs (lncRNAs) are defined as a class of RNA transcripts with length over 200 nucleotides and lack of protein-coding capacity, many of which exhibit specific cell-type and developmental-stage expression pattern [7, 8]. Emerging studies find that lncRNAs play crucial roles in a variety of cellular events, including transcriptional regulation of genes, cell proliferation, cell differentiation, cell cycle and apoptosis [9–11]. To date, mounting literatures have reported that the aberrant level of lncRNAs in numerous cancer is involved in carcinogenesis, tumor metastasis in diverse cancer types and can be considered as indicators for diagnosis and patient outcomes of cancer, such as prostate cancer, breast cancer, gastric cancer and CRC [12–16].

MicroRNAs are single-strand RNAs (18–22nt), which bind to seed sequences of 3′-untranslated regions (UTRs) of target genes to mediate translation inhibition [17]. LncRNAs exert their roles in genes expression network by affecting chromatin modification, mRNA transcription and interacting with RNA binding proteins [18–20]. In addition, lncRNAs also act as “miRNA sponges” and sequester miRNAs to modify miRNAs target genes transcription, which has been identified as the lncRNA-miRNA-mRNA regulatory network in cancer tumorigenesis and progression [21, 22]. For examples, LINC01287 regulates tumorigenesis of hepatocellular carcinoma via miR-298/MYB axis [23]. LncRNA SNHG15 promotes the proliferation and migration of lung cancer through targeting microRNA-211-3p [16]. LncRNA TUG1 sponges miR-145 to expedite cancer progression via modulating Sirt3/GDH axis [24].

Although a growing number of lncRNAs have been annotated in past decades, the role and potential regulatory mechanisms of uncharacterized lncRNAs in CRC still need to be clarified for exploration of potential diagnostic markers and therapeutic targets [25]. In the present study, we investigated lncRNAs expression profile in CRC by RNA sequencing (RNA-seq) based on Cancer Genome Atlas (TCGA) and characterized the role of long non-coding RNA LINC02418 as a novel oncogene in colon cancer. Mechanism analysis revealed that LINC02418 acted as competing endogenous RNA (ceRNA) for miR-34b-5p to prevent the degradation of BCL. Our results highlighted that LINC02418/miR-34b-5p/BCL2 axis might be a promising therapeutic target for CRC treatment.

## Materials and methods

### Clinical specimens

Tumor tissues and adjacent tissues from CRC patients (*n *= 20) were collected from China-Japan Union Hospital of Jilin University and all the participants signed the consent forms. Among the 20 patients, 11 patients were male and 9 patients were female. The average age of included patients was 59.75 ± 9.00 years old. The enrolled patients were pathologically diagnosed as CRC and did not undergo preoperative radiotherapy and/or chemotherapy prior to resection. The project was authorized by the Ethical and Scientific Committee of China-Japan Union Hospital of Jilin University. All samples were stored at − 80 °C until subsequent analysis.

### Cell culture

Human CRC cell lines including SW460, HCT116, HT-29, LoVo, Colo205, SW480 and normal colon epithelial cell line NCM460 were obtained from American Type Culture Collection (ATCC, Manassas, VA). Cells were cultured in RPMI 1640 medium supplemented with 10% fetal bovine serum (Gibco, Carlsbad, CA), 100U/ml penicillin and 100 mg/ml streptomycin. Cells were normally maintained at 37 °C in an incubator with 5% CO^2^ until use.

### Cells transfection

The sequence of BCL2 was inserted into vector pcDNA3.1 (Santa Cruz, Dallas, TX) for its ectopic expression in HCT116 and LoVo cell lines. Constructions, miR-34b-5p mimic, miR-34b-5p inhibitor and miR-NC mimic were delivered into CRC cells by Lipofectamine 2000 reagent (Invitrogen, Carlsbad, CA) according to manufactures instructions.

### Establishment of stable cell lines

Three shRNAs sequences targeting LINC02418 and negative control sequence were inserted into HuSH shRNA GFP Lenti Cloning Vector (Origene, Rockville, MD) following commercial guidance. The lentivirus was packaged using 293T cells following common protocols.

### Cell counting kit-8 analysis and colony formation assay

For CCK-8 experiment, the cells were seeded at density of 5 × 10^4^ cells per well on 48-well plate. Cells were harvested at 12 h, 24 h, 48 h and 72 h, and cell proliferation was assessed using cell counting kit-8 (Beyotime, Shanghai, CHA) according to the manufacturer’s instructions. The optical density (OD) at 450 nm was measured using a microplate reader (Thermo Fisher Scientific, Waltham, MA).

For colony formation assay. Total number of 3000 cells were seeded in 6-well plates and maintained in RPMI 1640 medium containing 10% FBS. After culture of 14 days, cells were fixed with 4% paraformaldehyde for 15 min and then stained with 0.1% crystal violet (Sigma-Aldrich, St. Louis, MO) for another 5 min till manually counting of visible colonies.

### Xenograft assay

Male BALB/c nude mice (5–6 weeks old) were maintained under specific pathogen-free facility and were manipulated according to protocols approved by the China-Japan Union Hospital of Jilin University. All the mice were randomly divided into four groups, and each group contained 5 mice.

HCT116-sh-LINC02418, HCT116-sh-NC, LoVo-sh-LINC02418 and LoVo-sh-NC cells (2 × 10^6^ cells) resuspended in 200 μl of medium were subcutaneously inoculated into nude mice. After 7 days post-injection, tumor size was measured every 3 days, and the tumor volumes were recorded. After 21 days post-injection, mice were sacrificed by cervical dislocation. Tumors were separated from mice and the weight of tumor was measured. The survival curve analysis was not involved in this experiment.

### Transwell migration and invasion assay

Cells migration assays were performed in a 24-well transwell chamber (CoStar, Badhoevedorp, Netherlands). Cells were plated and allowed to migrate through 8 μm-pore sized polycarbonate membrane. The chamber for invasion assay was pre-coated with 1 mg/ml Matrigel (Sigma-Aldrich, St. Louis, MO). A number of 5 × 10^4^ cells were added to the upper chamber of the transwells with FBS-free medium and the lower chamber was filled with 500 μl RPMI 1640 medium containing 10% FBS. After 24 h incubation, the cells were fixed by 4% formaldehyde for 10 min, stained by 0.1% crystal violet for 20 min. Images were captured under microscope.

### Quantitative reverse transcription PCR (qRT-PCR) assay

Total RNA was extracted from clinical tissue and CRC cells by TRIzol reagent (Invitrogen, CA, USA). RNA reverse transcription for mRNA and miRNA was performed using Prime ScriptTM RT Master Mix (Takara, Otsu, Japan) and TaqMan MicroRNA Reverse Transcription system (Thermo Fisher Scientific, MA, USA), respectively. The quantitative PCR was carried out by SYBR Premix Ex Taq II (TaKaRa Biotechnology, Dalian, China). Commercial miRNA qRT-PCR primers for miR-34b-5p and U6 were purchased by RiboBio (Guangzhou, China). The available primers sequences were as follows: LINC02418-F: 5′-ATTTCCATGGCGTTTCTCAC, LINC02418-R: 5′-AGGCAGGAGAATTGCTTGAA; BCL2-F: 5′-GGCATCTTCTCCTTC CAG-3′, BCL2-R: 5′-CATCCCAGCCTCCGTTAT-3′; GAPDH-F:5′-ACAACTTTGGTATCGTGGAAGG-3′, GAPDH-R:5′GCCATCACGCCACAGTTTC-3′. Applied Biosystems 7900 Real-Time PCR System (Applied Biosystems, Foster City, CA) was used for RNA quantification assay. U6 and GAPDH were used as internal control of miRNAs and mRNAs, respectively. Fold change was calculated by the 2^−△△Ct^ method.

### Luciferase assay

The fragments containing the binding sites or the mutated sites were synthesized and inserted into a pGL3-basic vector for dual luciferase assay. HCT116 and LoVo Cells were seeded in a 12-well plate and co-transfected with reporter plasmids and miR-NC/miR-34b-5p mimic. After 48 h, cells were harvested, dual-luciferase reporter assays were performed according to the protocol using a Dual-Luciferase Reporter Assay System (Promega, Madison, WI) on a GloMax 20/20 luminometer (Promega, Madison, WI).

### Fluorescence in situ hybridization (FISH) assay for miRNA

HCT116 and LoVo cells reaching 70% confluency were fixed in 4% paraformaldehyde for 20 min at room temperature, followed by permeabilized treatment in 70% ethanol at 4 °C overnight. For cellular miR-34b-5p detection, FISH assay was conducted following previous procedures [26]. Specific Digoxigenin (DIG)–labeled locked nucleic acid (LNA) probe against miR-34b-5p was purchased from QIAGEN (Hilden, Germany). The 4′,6-diamidino-2-phenylindole (DAPI) (Invitrogen, CA, USA) was adopted to stain the cell nucleic.

### Immunohistochemistry (IHC) assay

The tumor samples from CRC patients were fixed in 10% formaldehyde, embedded in paraffin and then sectioned into slices. For IHC assay, tumor slices were firstly deparaffinized and rehydrated. After washing, slices were treated with H_2_O_2_ to reduce the endogenous peroxidase activities. Slices were then incubated with primary antibody against BCL2 (1:50, Abcam, Cambridge, UK) overnight at 4  °C. With three times washing in PBS, the slides were incubated with secondary streptavidin–horseradish peroxidase-conjugated antibody (1:3000, Abcam, Cambridge, UK) for 1 h, and reacted with 3,3-diaminobenzidine tetrahydrochloride (DAB) solution (Yeasen Biotech, Shanghai, China) for 5 min. Finally, slides were counterstained with hematoxylin solution (Beyotime Biotechnology, Shanghai, CHN) for 1 min, dehydrated and mounted with neutral gum.

### Western blot

Protein samples were separated on 10% sodium dodecyl sulphate–polyacrylamide gel electrophoresis (SDS-PAGE) and then transferred to polyvinylidene fluoride membranes (PVDF) (Millipore, Billerica, MA) for protein bands detection. The membranes were blocked in 5% nonfat milk. Primary antibodies including anti-GAPDH (1:2000, Abmart, Shanghai, CHN), anti-BCL2 (1:500, Abcam, Cambridge, UK), anti-Caspase 9 (1:1000, Cell Signaling Techonolgy, MA, USA), anti-cleaved-Caspase 9 (1:500, Cell Signaling Techonolgy, MA, USA), anti-Caspase 3 (1:1000, Abcam, Cambridge, UK), and anti-cleaved-Caspase 3 (1:1000, Abcam, Cambridge, UK) were used for incubation overnight at 4 °C. The membranes were incubated with HRP-conjugated secondary antibody (1:3000, Jackson Immuno Research, PA, USA) at room temperature for 1 h and the protein bands were detected by Pierce Fast Western Blot Kit (Thermo Fisher Scientific, MA, USA).

### Flow cytometric analysis

Apoptosis assays of HCT116 and LoVo cells were performed using Annexin V-FITC/propidium iodide (AV/PI) Apoptosis Detection Kit (Abcam, Cambridge, UK) according to the commercial instruction. Cells were incubated with ice-cold 75% ethanol at 4 °C overnight, followed by resuspending in 500 µl of 1 × Annexin V Binding Buffer. Then, cells were stained with 5 μl Annexin V/FITC and 5 μl Propidium Iodide (PI) in the dark for 15 min at the room temperature. Apoptosis rates were examined and analyzed by flow cytometry using FACS Calibur (BD Biosciences, CA, USA).

### Statistical analysis

Statistical data analysis was conducted using GraphPad Prism 6.0 (GraphPad Software, Inc., La Jolla, CA). Experiments were carried out in triplicate and the data was displayed as mean ± SD. Statistical analysis was conducted using Student’s *t* test or one-way analysis of variance. Statistical significance was considered when p < 0.05.

## Results

### LINC02418 expression is upregulated and associated with poor prognosis in CRC patients

**Fig. 1:**
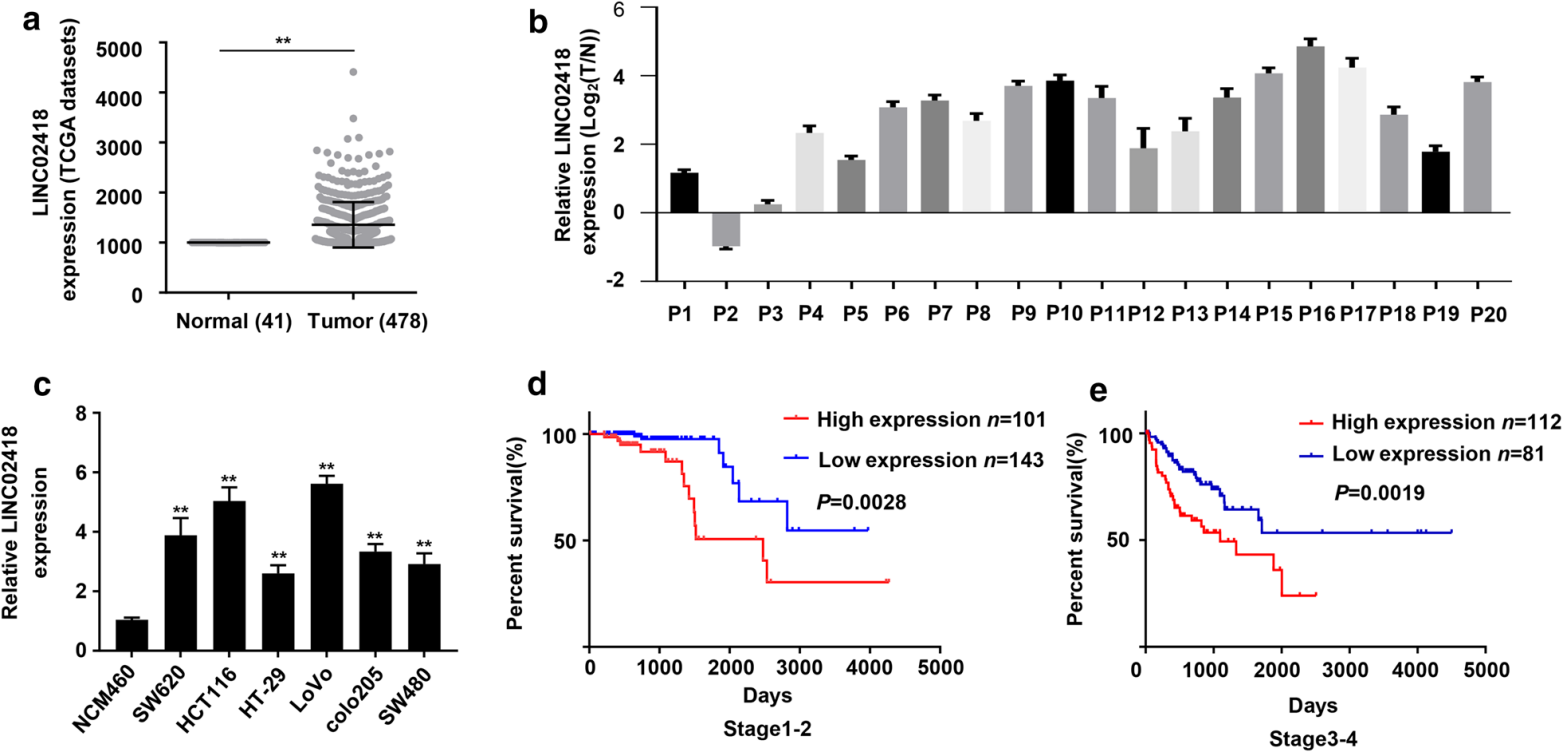
LINC02418 expression in CRC tissues and cell lines. **a** The differential expression of LINC02418 in CRC samples (*n *= 478) and adjacent normal colon tissues (*n *= 41) was analyzed based on TCGA; **P < 0.01, compared to adjacent tissue group. **b** Quantification analysis of LINC02418 level was conducted in 20 pairs of CRC samples and adjacent normal colon tissues. **c** LINC02418 levels in SW620, HCT116, LoVo, Colo205, HT-29, SW480 and normal colon epithelial cell line NCM460 were determined by qRT-PCR; **P < 0.01, compared to NCM460. **d** Kaplan–Meier analyses of the correlations between expression of LINC02418 and overall survival of patients with CRC in stage 1–2 and stage 3–4 (stage 1, 76 patients; stage 2, 168 patients; stage 3, 127 patients; stage 4, 66 patients). The error bars stand for standard deviation (SD). Data were representatives of three independent experiments with similar results

To identify the lncRNAs expression profile in CRC patients, we examined the expressional level of lncRNAs in human CRC samples and normal intestinal tissue. By using the Cancer Genome Atlas (TCGA) database, we found LINC02418 abundance was significantly up-regulated in CRC samples (*n *= 478) when compared with normal tissues (*n *= 41) (Fig. 1a).To validate the results, LINC02418 level in 20 pairs of CRC samples and adjacent tissues were examined by RT-qPCR. Similarly, quantification test showed LINC02418 was highly expressed in 19 out of the 20 CRC patients (Fig. 1b). These results implied that expression of LINC02418 in CRC tissues was markedly higher than that in normal tissues. Moreover, the expression of LINC02418 is increased by 2–6 folds in SW620, HCT116, LoVo, Colo205, HT-29 and SW480 cell line when compared with normal colon epithelial cell line NCM460 (Fig. 1c). HCT116 and LoVo cell lines were selected for further experiments, because of the highest expression level of LINC02418.

Next, to study the clinical significance of LINC02418 in CRC, the correlation between LINC02418 expression and prognosis was assessed by Kaplan–Meier survival assays using 437 patients information collected from TCGA database. As shown in Fig. 1d and e, in both early (Stage 1–2) and late stages (Stage 3–4), CRC patients with high expressional level of LINC02418 had poorer overall survival (OS) rates, indicating that LINC02418 was one potential indicator for prognosis prediction, and LINC02418 may promote CRC progress.

### Knockdown of LINC02418 inhibits tumor growth, cell mobility and cell invasion

**Fig. 2:**
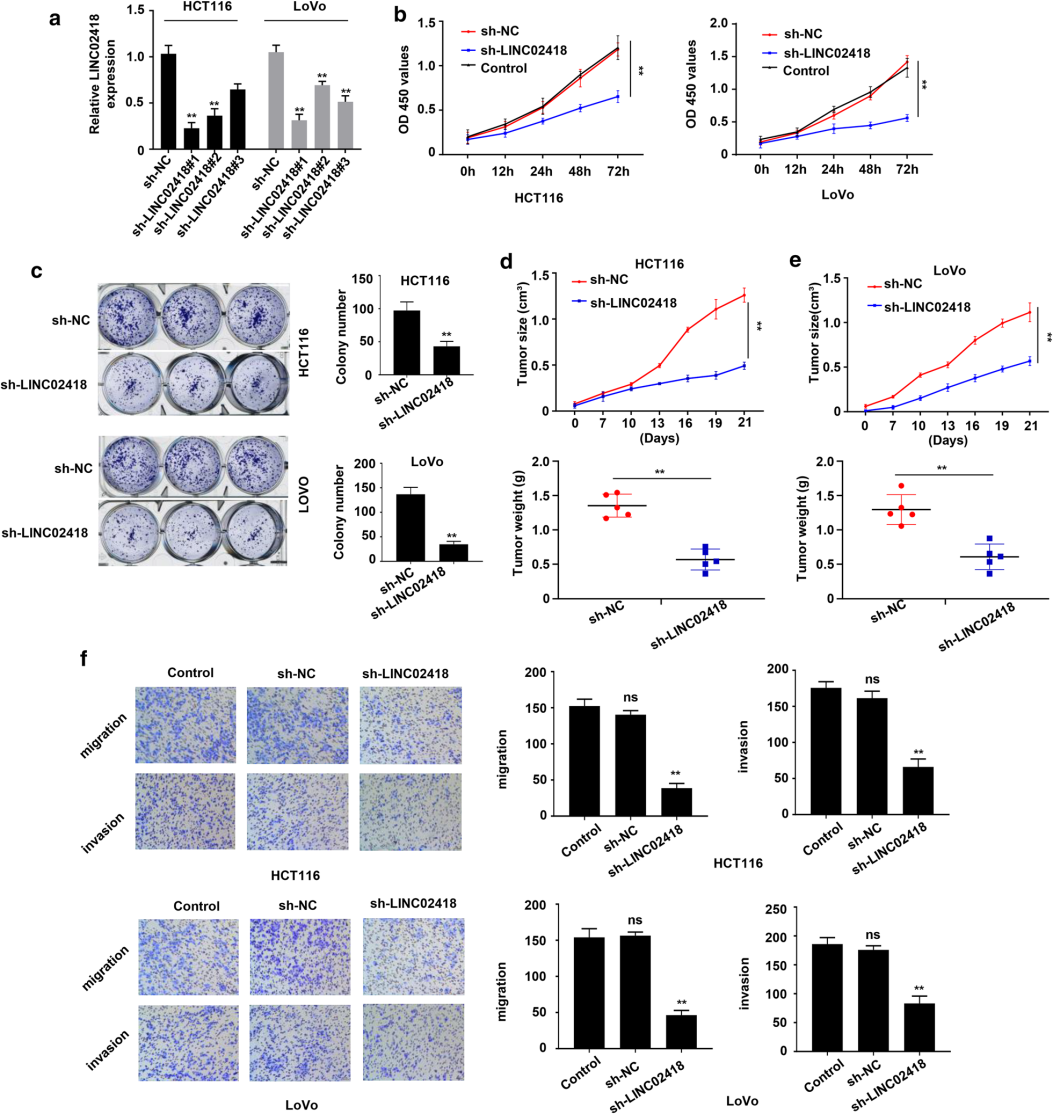
Down-regulation of LINC02418 inhibited tumor growth, cell mobility and cell invasion. **a** HCT116 and LoVo cells were transfected with three shRNAs targeting LINC02418 and control sh-NC. The cells were harvested at 48 h post transfection, and qRT-PCR was conducted to measure LINC02418 level. **b**, **c** Cell proliferation of HCT116 and LoVo cells were determined via CCK-8 assays (**b**) and colony formation assays (**c**). **d**, **e** Tumor volume curve and tumor wet weight of mice subcutaneously injected with HCT116 (**d**) and LoVo (**e**) cells which were stably transduced with sh-LINC02418 or sh-NC. **f** Transwell assay were performed to determine the effect of LINC02418 on the migration and invasion ability of HCT116 and LoVo cells. The results were expressed as the number of invaded cells per field. The error bars stand for standard deviation (SD). Data were representatives of three independent experiments with similar results; **P < 0.01, compared to sh-NC group; ns, no difference, compared to control group

In order to dissect the effect of LINC02418 on the biologic activity of CRC cell lines, HCT116 and LoVo cells with reduced LINC02418 levels were established by lentivirus infection. Based on the endogenous level of LINC02418, HCT116-sh-LINC02418#1 and LoVo-sh-LINC02418#1 were selected for further analysis (Fig. 2a). The CCK-8 assay revealed that compared with sh-NC transduced CRC cells group and blank control (without shRNA transducing) group, stable knockdown of LINC02418 statistically inhibited the proliferation of HCT116 and LoVo cells (Fig. 2b). The colony formation assay was carried out to further analyze the effect of LINC02418 on tumor cell growth. As revealed in Fig. 2c, stable down-regulation of LINC02418 significantly reduced the proliferation of HCT116 and LoVo cells. To evaluate whether LINC02418 affected CRC growth in vivo, subcutaneous tumor formation experiments were set up in nude mice. HCT116 and LoVo cells transduced with sh-LINC02418 or sh-NC were subcutaneously injected into the nude mice for 21 days. The tumor size and tumor weight were recorded at indicated times. As shown in Fig. 2d, e and Additional file 1: Fig. S1, tumor size and tumor weight of xenograft tumors from sh-LINC02418-transduced HCT116 and LoVo cells were obviously less than those from CRC cells transduced with sh-NC, which was consistent with the results of in vitro experiments. The transwell experiments showed that inhibition of LINC02418 significantly repressed mobility and invasiveness of HCT116 and LoVo cells (Fig. 2f). Taken together, these data suggested that LINC02418 contributed to CRC cell growth and metastasis both in vivo and in vitro.

### LINC02418 acts as a ceRNA for miR-34b-5p in CRC cells

**Fig. 3:**
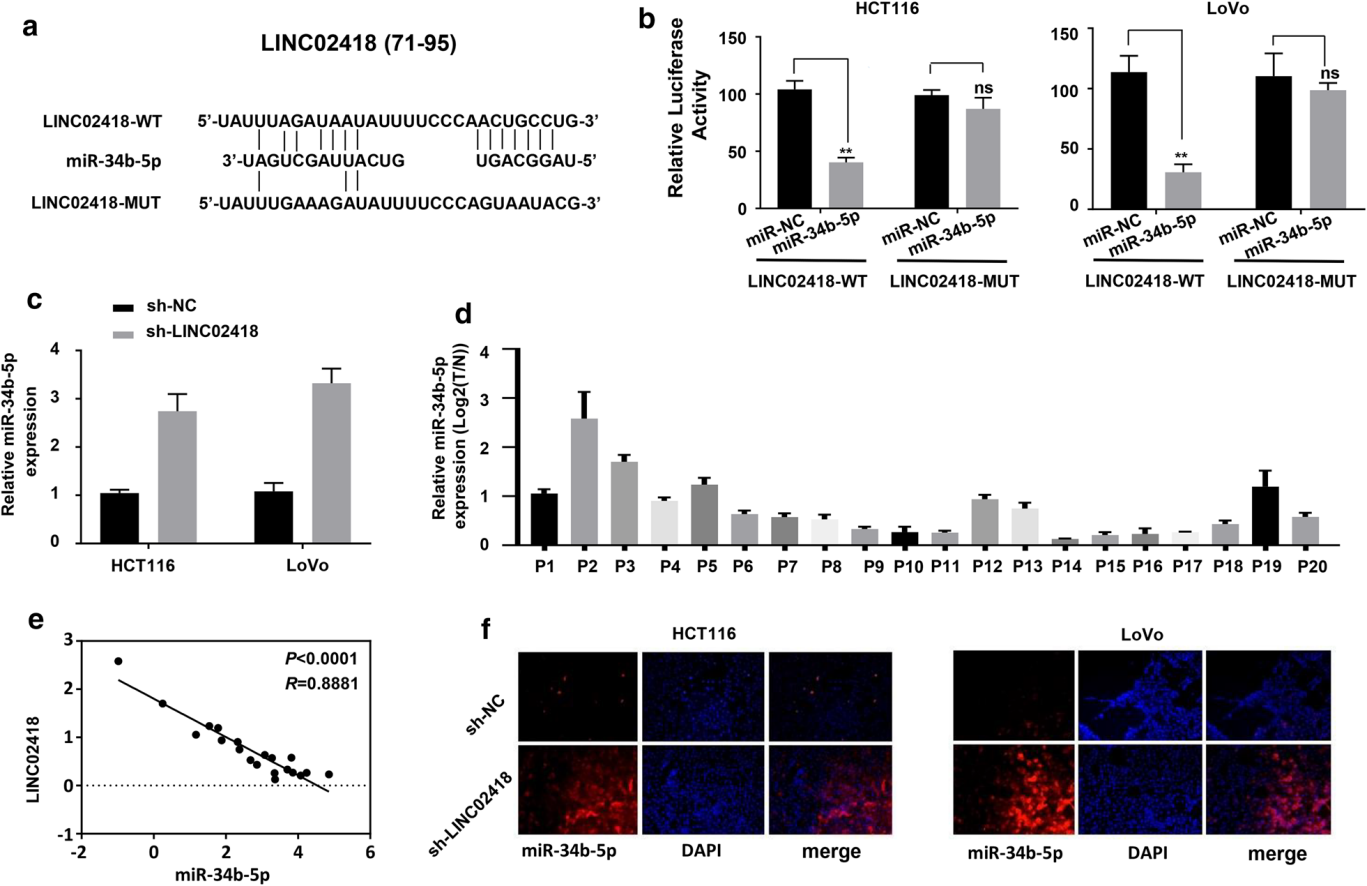
LINC02418 was a ceRNA for miR-34b-5p in CRC cells. **a** The putative binding sites of miR-34b-5p to wild type LINC02418 (LINC02418-WT), and the mutant LINC02418 (LINC02418-MUT). **b** Luciferase activities of HCT116 and LoVo cells co-transfected with miR-34b-5p mimic/miR-NC and luciferase reporters harboring LINC02418-WT or LINC02418-MUT were measured by dual luciferase assays; **P < 0.01; ns, no difference, compared to miR-NC group. **c** The effect of knockdown of LINC02418 on miR-34b-5p expression in CRC cells were quantified by qRT-PCR; **P < 0.01, compared to sh-NC group. **d** The differential expression of miR-34b-5p in CRC and normal tissues were analyzed by qRT-PCR. **e**, **f** The negative correlation between miR-34b-5p and LINC02418 levels was presented and determined by Pearson’s correlation curve and FISH experiments, respectively. The error bars stand for standard deviation (SD). Data were representatives of three independent experiments with similar results

It is documented that lncRNAs can exert their function trough acting as ceRNA of miRNAs [27]. The lncRNA-miRNA-mRNA regulatory network has been shown to be involved in the treatment of malignant tumors. To assess whether LINC02418 interacted with other miRNAs in CRC, the potential binding partner for LINC02418 was analyzed by online software StarBase v2.0. As shown in Fig. 3a, LINC02418 contained putative binding sequence for miR-34b-5p.

To confirm the binding between LINC02418 and miR-34b-5p, dual luciferase assays were performed in HCT116 and LoVo cell lines. The luciferase activity was markedly inhibited when LINC02418-WT and miR-34b-5p mimic were co-transfected into HCT116 and LoVo cells. Co-transfection of LINC02418-WT and miR-NC or co-transfection of LINC02418-MUT and miR-34b-5p mimic had no significant impact on luciferase activity (Fig. 3b). In addition, knockdown of LINC02418 in HCT116 and LoVo cells greatly enhanced the expression level of miR-34b-5p (Fig. 3c). Taken together, it could be found that LINC02418 negatively regulated miR-34b-5p expression in CRC cells.

Subsequently, we detected the relationship between LINC02418 and miR-34b-5p in clinical samples. In 20 pairs of CRC samples, LINC02418 expressional level negatively correlated with miR-34b-5p level (Fig. 3d, e). Moreover, fluorescence in situ hybridization (FISH) experiment showed that compared with CRC cells transduced with sh-NC, CRC cells transduced with sh-LINC02418 expressed higher level of miR-34b-5p, which provided evidence for the negative correlation between LINC02418 and miR-34-5p (Fig. 3f).

### BCL2 is the target of miR-34b-5p in CRC cells

**Fig. 4:**
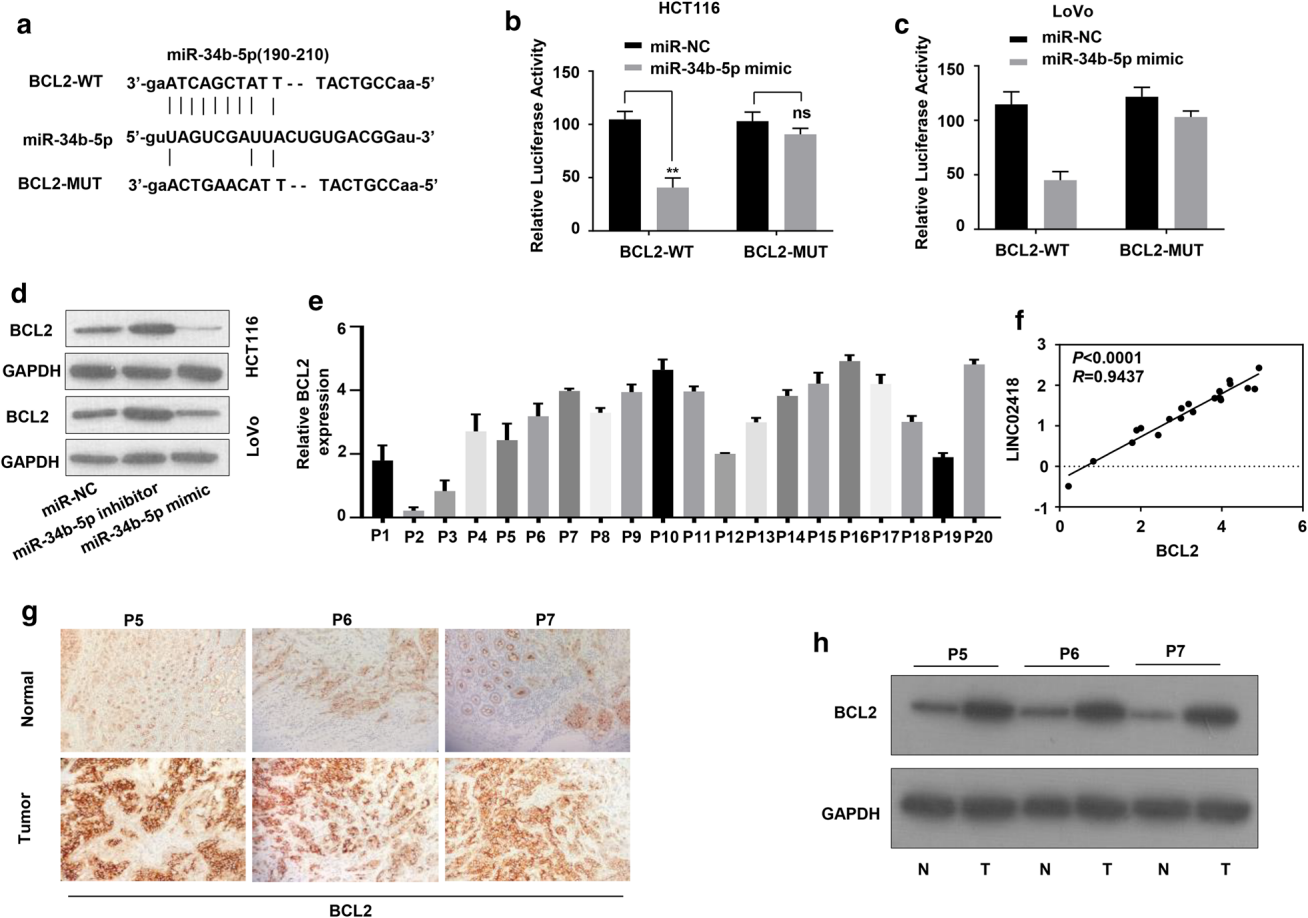
BCL2 was the target of miR-34b-5p in CRC cells. **a** The putative binding sites of miR-34b-5p to wild type *BCL2* (BCL2-WT) and the mutant *BCL2* (BCL2-MUT). **b**, **c** Luciferase activity detection of HCT116 (**b**) and LoVo (**c**) cells co-transfected with miR-34b-5p mimic/miR-NC and luciferase reporters expressing BCL2-WT or BCL2-MUT. **d** Endogenous protein level of BCL2 in HCT116 and LoVo cells transfected with miR-NC, miR-34b-5p inhibitor or miR-34b-5p mimic were detected by western blotting. **e** The expressional level of *BCL2* mRNA in CRC and normal tissues were examined by qRT-PCR. **f** Pearson’s correlation curve revealed the positive relevance between *BCL2* and LINC02418 levels. **g**, **h** IHC and WB assays were performed to detect the protein level of BCL2 in CRC and normal tissues. The error bars stand for standard deviation (SD). N, normal tissue; T, tumor tissue. Data were representatives of three independent experiments with similar results; **P < 0.01; ns, no difference, compared to miR-NC group

BCL2, the gatekeeper for cell apoptosis, was identified as one possible target for miR-34b-5p by using software TargetScan (Fig. 4a). Interestingly, previous articles reported that miR-34b-5p regulates multiple cellular processes including cell apoptosis and cell proliferation through participating in a several critical signal pathway like VEGF-A, BCL2 and Notch [28]. To determine the interaction between *BCL2* and miR-34b-5p, wild type 3′UTR (containing miR-34b–5p recognition site) and the mutant 3′UTR of *BCL2* were sub-cloned into luciferase reporter plasmids. Dual-luciferase assays showed that miR-34b-5p mimic transfection reduced luciferase activity in wild type construction but not in mutant type construction (Fig. 4b, c). In parallel, miR-34b-5p transfection reduced endogenous protein level of BCL2, while the addition of miR-34b-5p inhibitor significantly restored BCL protein expression in both HCT116 and LoVo cells (Fig. 4d). These results proved that BCL2 was one of the target genes of miR-34b-5p. Quantification of *BCL2* in 20 pairs of CRC samples revealed that BCL2 expressional level was positively correlated with the amount LINC02418 (Fig. 4e, f). We randomly selected 3 pairs of CRC samples for further confirmation. As shown in immunohistochemical (IHC) and western blot assays, the protein level of BCL2 was greatly increased in CRC samples from patients (Fig. 4g, h).

### LINC02418 promotes colon cancer cell proliferation by upregulating BCL2 via sponging miR-34b-5p

**Fig. 5:**
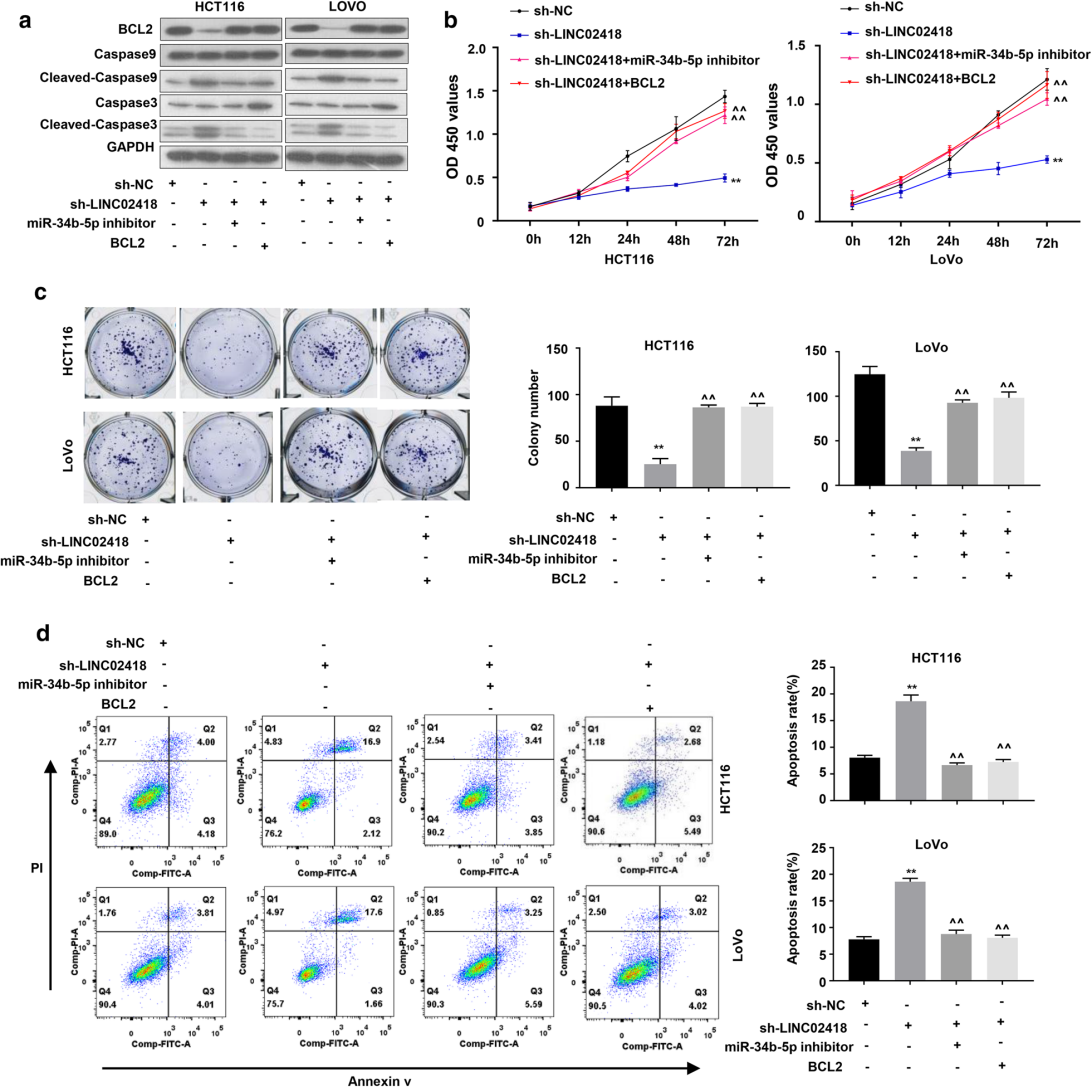
LINC02418 indirectly regulated BCL2 expression through sponging miR-34b-5p. **a** Endogenous protein level of BCL2, caspase 9, caspase 3 and cleaved-caspase 9, cleaved-caspase 3 were detected in HCT116 and LoVo cells by western blotting. **b**, **c** Cell growth of HCT116 and LoVo cells were determined by CCK-8 assays and colony formation assays. **d** Apoptosis rates of HCT116 and LoVo cells were analyzed by flow cytometry assays. Each independent experiment contained 3 repeated samples per group. The error bars stand for standard deviation (SD). Data were representatives of three independent experiments with similar results; **P < 0.01, compared to sh-NC group; ^^^^*P *< 0.01, compared to sh-LINC02418 group

To elucidate the effect of LINC02418 on miR-34b-5p/BCL2 axis, protein level of several apoptotic markers were detected in HCT116 and LoVo cells. Western blot analysis showed that knockdown of LINC02418 greatly enhanced the amount of cleaved-Caspase 9 and cleaved-Caspase 3, and strongly reduced BCL2 expression. Transfection of miR-34b-5p inhibitor into cells with defective expression of LINC02418 not only reduced the level of cleaved forms of Caspase 9 and Caspase 3, but also promoted protein expression of BCL2. Overexpression of BCL2 also reduced protein level of cleaved-Caspase 9 and cleaved-Caspase 3 (Fig. 5a). Subsequently, CCK-8 and colony formation assays showed inhibition of LINC02418 repressed cell growth in HCT116 and LoVo cells. However, transfection of miR-34b-5p inhibitor or overexpression of BCL2 abolished the effect of down-regulated LINC02418 on cell proliferation (Fig. 5b, c). Inversely, knockdown of LINC02418 dramatically elevated the apoptosis rate of CRC cells while addition of miR-34b-5p inhibitor and BCL2 reduced insufficient LINC02418 expression-promoted cell apoptosis (Fig. 5d). These findings demonstrated that LINC02418 regulated colon cancer cell proliferation through upregulating BCL2 expression via sponging miR-34b-5p.

## Discussion

Colon cancer is one common malignancy of digestive tract and has been one of the most serious healthy threaten worldwide. There is urgent need to explore more effective early diagnostic indicators and treatment strategies.

In this study, with analyzing of TCGA database, we found that LINC02418 level was up-regulated in CRC samples and cell lines, and was closely associated with prognosis of CRC (Fig. 1). Subsequently, we identified the effect of LINC02418 on CRC cells growth, migration and invasion (Fig. 2). Further bioinformatic screening and dual-luciferase assays showed that miR-34b-5p could bind to LINC02418 and BCL2 gene, and was negatively correlated with the amount of LINC02418 (Fig. 3 and Fig. 4). Finally, cell growth and colony formation experiments displayed that LINC02418 regulated CRC cells proliferation by regulating BCL2 via sponging miR-34b-5p (Fig. 5).

Long non-coding RNAs and microRNAs play multiple roles in tumor progression in almost all organs. The specificity expression in tissue and cells and high throughput detection technology make lncRNAs and miRNAs become potential diagnostic and therapeutic targets for clinical treatment [7, 21, 22, 25]. In CRC patients, the high expression of LINC02418 correlated with poor prognosis of CRC and negatively correlated with the amount of miR-34b-5p, indicating LINC02418 abundance could be used as candidate indicator for CRC diagnosis and prognosis. If the expression of LINC02418 can be analyzed together with other clinical indicators, such as gender and age for comprehensive analysis, it may be possible to improve the accuracy of LINC02418 as a predictor in CRC.

Knockdown of LINC02418 decreased the CRC cell growth in vitro and in vivo and limited cell migration and invasion ability, indicating that LINC02418 was able to promote CRC progress and might have a vital role in regulating tumor development-associated signaling pathways. MicroRNAs are a group of small non-coding RNAs which bind to cognate mRNA via base pairing principles and decrease the expression of target gene either by translational repression or mRNA degradation [17]. Profound evidence suggests that miRNAs are dysregulated in a variety of cancer tissues and may play distinct roles according to the type of cancer, disease stage, or the molecules that interact with it [29–32].

Through competitively binding to microRNAs, lncRNAs attenuate the regulatory effect of microRNAs on target genes. The lncRNA/microRNA/mRNA network has already been proved to be critical for cancer occurrence and development. Bioinformatic analysis revealed that LINC02418 and 3′UTR region of *BCL2* contained complementary sequence of miR-34b-5p, implying miR-34b-5p could bind to LINC02418 and *BCL2* gene. MiR-34b-5p belongs to the miR-34 family which has been reported to be tumor suppressor and therapeutic candidate in cancer [33–36]. Dual-luciferase activity assays confirmed the interaction between miR-34b-5p and LINC02418 (Fig. 3) or 3′UTR region of *BCL2* (Fig. 4). Moreover, quantification assays determined that expressional level of miR-34b-5p was negatively correlated with the amount of LINC02418 and *BCL2* in CRC patients (Fig. 3), indicating the regulatory function of LINC02418/miR-34b-5p/BCL2 axis in CRC.

BCL2 is believed to suppress apoptosis in a variety of tissues and cancers. BCL2 inhibits the release of cytochrome c and pro-apoptotic factors, so that the relevant factors are not able to initiate the downstream caspase pathway to activate Caspase 9 and Caspase 3 [37]. The positive correlation between LINC02418 and BCL2 expression level suggested dysregulation of apoptosis might be associated with the contribution of LINC02418 in CRC progression. Western blot experiments showed in the presence of sh-LINC02418, protein level of cleaved-Caspase 9 and cleaved-Caspase 3 in CRC cells was significantly increased while BCL2 expression was inhibited, indicating silence of LINC02418 could improve cell apoptosis and reduce colon cancer cells growth (Fig. 5a). However, the protein level of BCL2 in cells with decreased LINC02418 was restored by miR-34b-5p inhibitor transfection. The CRC cells growth was also compensated either by down-regulation of mir-34b-5p or ectopic expression of BCL2 protein (Fig. 5b, c).

Combining all the evidence from the study, we speculated that in normal intestinal epithelial tissue, LINC02418 stays in a low level which leads to the expression of miR-34b-5p as well as low expression of BCL2. As a consequence, cell apoptosis is activated. However, in human CRC cells, LINC02418 expression is upregulated and the expression of miR-34b-5p and BCL2 are affected by increased level of LINC02418. Thus, cell apoptosis is inhibited, allowing cancer cells escaping from cell death and re-entering abnormal cell cycles.

## Conclusion

Briefly, our present study demonstrated LINC02418 was upregulated in colon cancer, which promoted tumor cells growth and migration. LINC02418 could bind to miR-34b-5p to sequester the binding between miR-34b-5p and its target gene *BCL2*. Hence, LINC02418 positively regulated tumorigenesis through LINC02418/miR-34b-5p/BCL2 axis and might be indicated as a biomarker for CRC.

## Supplementary information

**Additional file 1: Fig. S1.** Representative images of xenograft tumors separated from 4 groups of mice subcutaneous injected with HCT116-sh-NC, HCT116-sh-LINC02418, LoVo-sh-NC, LoVo-sh-LINC02418 cells.

## Data Availability

The datasets used during the present study are available from the corresponding author on reasonable request.

## Abbreviations

lncRNAs: Long non-coding RNAs
CRC: Colorectal cancer
miRNA: microRNA
CCK-8: Cell Counting Kit-8
BCL2: B-cell lymphoma-2 (BCL2)
ceRNA: Competing endogenous RNA
