# Supplementary figures and images for: LINC02418 promotes colon cancer progression by suppressing apoptosis via interaction with miR-34b-5p/BCL2 axis
Source: Cancer Cell Int. 2020 Sep 22;20:460. doi: 10.1186/s12935-020-01530-2 (PMC7507712; doi:10.1186/s12935-020-01530-2)

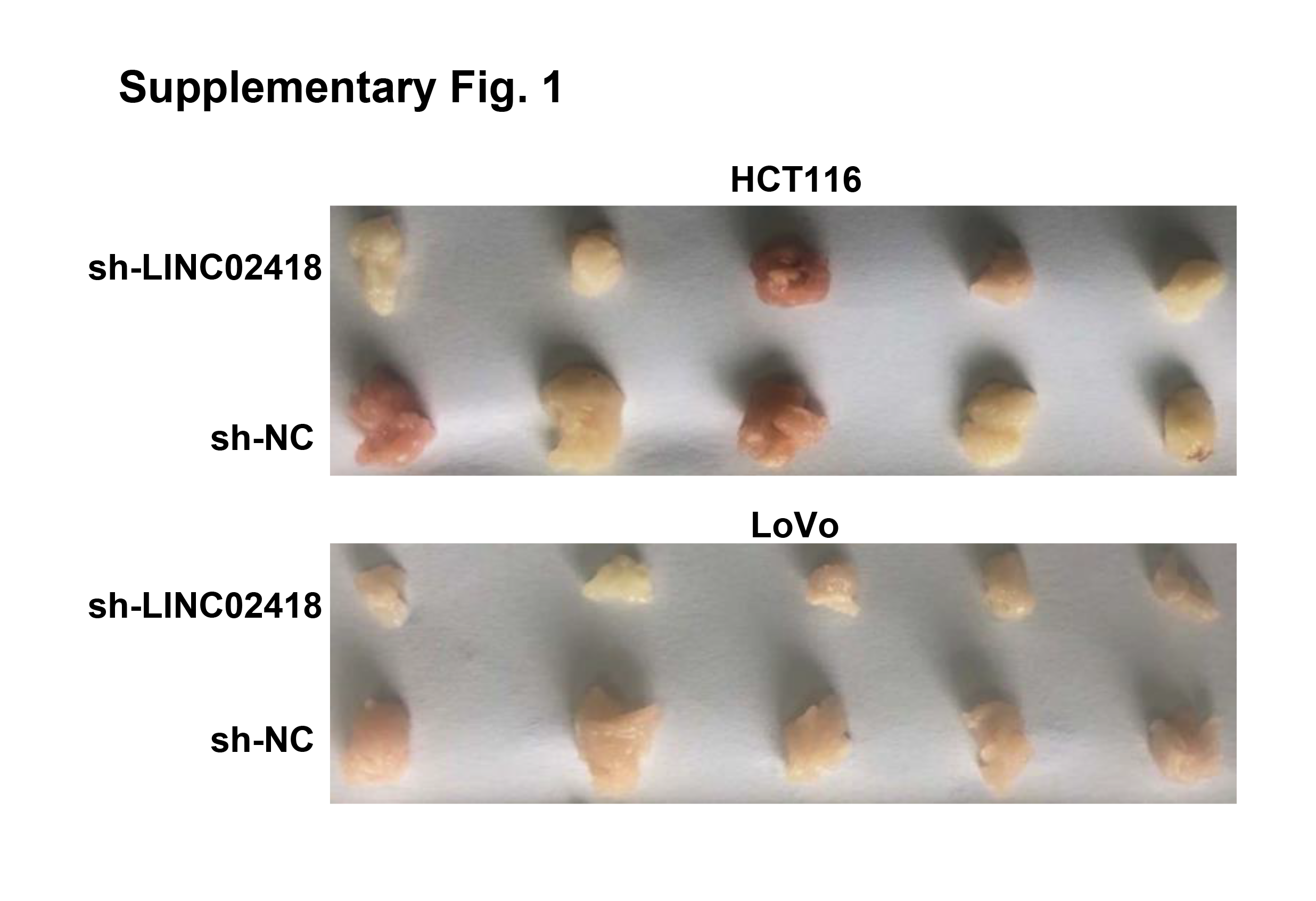

Supplement: Supplementary file 1 — Additional file 1: Fig. S1. Representative images of xenograft tumors separated from 4 groups of mice subcutaneous injected with HCT116-sh-NC, HCT116-sh-LINC02418, LoVo-sh-NC, LoVo-sh-LINC02418 cells. [file 12935_2020_1530_MOESM1_ESM.tif]
